# Actual Use Behavior Assessment of a Novel Puff Recording Electronic Nicotine Delivery System: Observation Study

**DOI:** 10.2196/43175

**Published:** 2023-02-08

**Authors:** Xiang Gao, Liam Humberstone, Yatao Liu

**Affiliations:** 1 Scientific Horizons Consulting Irvine, CA United States; 2 Totally Wicked Limited Blackburn United Kingdom

**Keywords:** e-cigarettes, electronic nicotine delivery system, behavior assessment, nicotine addiction, PR-ENDS

## Abstract

**Background:**

Compared with combustible cigarettes, electronic cigarettes (e-cigarettes) can deliver a sufficient amount of nicotine with a significantly reduced emission of toxicants, which renders them as potential harm reduction candidates for tobacco and smoking replacement. However, the use of e-cigarettes is not harm free and the long-term health effect of using e-cigarettes is yet to be established. Given the high prevalence of e-cigarette use across the globe and its potential health concerns, it is imperative to conduct actual use behavior assessments to better understand how e-cigarettes are being consumed in real-world conditions. However, with the currently available technologies, there is still a lack of noninvasive, noninterventional, and convenient instruments for the real-time and real-world use behavior monitoring of e-cigarette product use. Novel technology-based systems that do not primarily rely on self-report or intrusive measurements to monitor e-cigarette use behaviors are therefore highly desired.

**Objective:**

The primary goal of this study is to investigate the e-cigarette actual use behaviors in the real world via a novel puff recording electronic nicotine delivery system (PR-ENDS). Specifically, we aim to analyze and summarize the survey and PR-ENDS use data and to study the relationships and effects of different factors on these variables.

**Methods:**

In real-world conditions, 61 enrolled UK e-cigarette users were instructed to use PR-ENDS as the primary source of nicotine with their selected e-liquids for at least 3 weeks (21 days). A baseline survey was conducted to collect information about participants’ demographics and nicotine use history (cigarette and ENDS). The puff data (ie, puff number, puff duration for each puff, device power, e-liquid nicotine concentrations) were directly recorded by PR-ENDS and uploaded to the cloud for further analyses. The nicotine emission and nicotine consumption were estimated based on recorded puff data.

**Results:**

Middle-aged adults with a nicotine history represented the major user profile during the PR-ENDS trial. A wide range of device power and e-liquid nicotine concentrations was applied and their combinations during actual use were found to be rather complex. Various puff parameters (ie, puff duration, puff number, nicotine emission) were assessed with contributing factors from device, e-liquid, and user nicotine history in different effect sizes. The real-time observation revealed substantial intra- and interindividual variabilities in PR-ENDS use behaviors. The use pattern of a quick adaptation followed by consistent product use was recognized for at least 3 weeks during actual use.

**Conclusions:**

The actual use behavior assessment of PR-ENDS was conducted as a proof-of-concept application. The complex interactions of product attributes and significant intra- and interindividual variabilities in e-cigarette use behaviors provided new insights of compensatory behavior, which can inspire future studies in the field of nicotine addiction and abuse liability behavior assessment.

## Introduction

Regardless of the increasing prevalence of electronic cigarettes (e-cigarettes) worldwide, little is known about how these products are being used in the real world [1]. Indeed, it is essential to conduct “actual use” behavior assessments to better understand how e-cigarettes are being consumed in real-world conditions. From individual health perspectives, the harmful emissions and abuse liability associated with e-cigarette use [2-4], as well as their variabilities within and between different user groups, still need to be quantitatively evaluated. Product-specific puff topography and puffing behavior are still an area where there is a dearth of reliably recorded data. From a public health perspective, being able to closely monitor the group-level use pattern of e-cigarettes (eg, frequency, intensity, use over time) associated with product attributes (ie, device power, e-liquid nicotine concentration) can effectively inform a regulatory body on product use trends. In this way, any early signs of use inappropriate for the protection of public health can be mitigated or even avoided beforehand.

Until now, many efforts have been made toward assessing the actual use behaviors of e-cigarettes [5-13]. However, questions remain in this field, with knowledge gaps present in numerous aspects. First, most e-cigarette use behavior research to date has focused on products that have preset attributes, in which users cannot freely change e-cigarette settings (eg, power and e-liquid) during the investigation. However, in an actual use scenario, users might change their preferences over time, selecting different product settings and e-liquids (ie, nicotine strength) over time. This is especially true for those who aim to cut back nicotine consumption and cease nicotine use entirely over time. This could become important in demystifying whether there is any “sweet spot” in combinations of product attributes for different users. Another concern to address in e-cigarette actual use behavior assessment is whether the “self-titration” and puff compensatory effect still applies when there is more than 1 factor that can influence users’ puffing behavior. As pointed out in a previous study [14], experienced e-cigarette users tend to puff more frequently and longer when using e-liquids with a low nicotine concentration. Yet, when the impact of e-liquid is compounded by device power, the compensatory behavior may become less definitive. Authors from another study [8] indicated that device power might also trigger a compensatory effect and change users’ puffing behavior. During the actual use of e-cigarettes, the interactions between device, e-liquid, and puff topography are rather complex, and the effect of different factors on puffing behaviors is still largely unknown. Furthermore, the puff topography and puffing behaviors of e-cigarette use often dynamically evolve over time at individual and population levels. However, previous studies mostly reported study durations lasting from a few days to a week [6,10,12,13], which is a relatively short period compared with actual use situations, and therefore limited researchers’ capabilities in observing any extended product use behaviors over a longer term. Thus, it is important to investigate the intra- and interindividual as well as population behavior consistency or variability during the actual use of e-cigarettes and for a time span of a few weeks or even longer.

To conduct actual use behavior assessment of e-cigarettes in the real world, the ideal practice should incorporate several considerations. First, the assessment needs to be conducted in a noninvasive and noninterventional fashion, as e-cigarette users under managed observation tend toward behavior different from that in habitual use [11,15]. Second, during the observation, the product use information should be collected objectively and analyzed in real time. In this case, any specific product use pattern or trend can be identified in a timely manner, without the delay of follow-up retrospective analyses or any subjective recall bias. Third, the monitored products should be easily accessible to groups with diverse demographics for a relatively long time span [7]. Only by conducting real-time longitudinal observations can real-world e-cigarette use behavior and associated patterns over time be specifically identified.

Various methods have been applied to monitor the puff topography and puffing behavior during the actual use of e-cigarettes. However, limitations still exist. These include recall and response bias [16-19], unnaturalistic behaviors under training and investigations [20], high cost and time consumptions [21-23], and unavoidable intervention on aerosol transportation in mouthpiece adaptors [24]. Recently, a novel puff recording electronic nicotine delivery system (PR-ENDS) (VITRO [Registered Trademark], Shenzhen JWEI Electronics Co, Ltd) was reported [25] with the demonstration of its feasibility in measuring naturalistic puff topography as well as estimating nicotine consumptions during the ad libitum use. PR-ENDS is an open refillable device with a removable 0.8-ohm coil, 2 mL e-liquid capacity, and 3 power output settings (low power: 7-9 W, medium power: 9-11 W, and high power: 11-13 W). One unique design feature of PR-ENDS is that it is able to measure puff parameters such as the number of puffs, puff duration, and puff intervals through a built-in chip in the device. The recorded data can then be uploaded to the cloud in real-time via a smartphone or computer-based app, which is similar to the concept of “internet of things” for personal wearable devices [26,27]. The PR-ENDS device uses information such as e-liquid nicotine concentration, propylene glycol–to–vegetable glycerin ratio (ie, PG-to-VG ratio), e-liquid brands, device power that can be simultaneously reported and obtained in situ and integrated with the recorded puff topography data to approximate device nicotine emission puff by puff. Further investigation of the pharmacokinetic profiles of PR-ENDS ad libitum use via blood sample analysis has shown that the device is capable of estimating inhaled nicotine intake based on the comparability of PR-ENDS–calculated nicotine consumption and PR-ENDS–measured nicotine concentration in blood specimens [25]. By connecting PR-ENDS with a personal mobile device, such as a smartphone via securely paired Bluetooth, users can view their own puffing data and track their own consumption of nicotine in real time. This is especially helpful for those who want to gain awareness of their nicotine consumption to help quit or cut back on nicotine use.

The aforesaid design features of PR-ENDS allow us to conduct real-time, actual use behavior assessment to investigate device-specific puff topography and puffing behaviors in a naturalistic manner at both individual and population levels. In this report, we investigate real-world and real-time e-cigarette use behaviors with the help of data collected from PR-ENDS devices. Specific objectives focus on (1) investigating the usage of PR-ENDS with combinations of different device powers and e-liquids; (2) assessing puff topography and puffing behaviors (ie, puff duration, puffs per day, nicotine emission per puff) of the observed cohort during actual use; (3) quantifying the correlation effects between puff topography and PR-ENDS–specific product attributes and user nicotine profile; (4) exploring real-time behavior patterns of PR-ENDS users during actual use and over a period of 3 weeks and beyond.

## Methods

### Participants and Procedures

The actual use behavior assessment of PR-ENDS includes observing a single group of e-cigarette users enrolled during a product trial study in the UK via vape shops. In total, 61 participants agreed to participate in the assessment, with a requirement of using the PR-ENDS device as their primary source of nicotine with their selected e-liquids for the trial duration. Additional inclusion criteria include (1) older than 18 years (UK minimum legal age to smoke); (2) does not have a history of chronic disease or psychiatric condition; (3) does not regularly use prescription medication; (4) not pregnant; and (5) not enrolled in a smoking cessation program. The recruited participants were instructed to use the PR-ENDS device together with a Bluetooth paired smartphone/computer-based app from the beginning of the product trial. A baseline survey was conducted to collect information about participants’ demographics and nicotine use history (cigarette and ENDS). Only the unique device ID was used to differentiate users, and no personally identifiable information was collected. Participants consented to release the survey and puff topography data for marketing research, regulatory submission, and publications.

After completing the baseline survey, participants were instructed to use the PR-ENDS device in real-world conditions for 3 weeks (21 days). At the end of the third week, a follow-up survey was conducted to collect information about any adverse events experienced by participants. If no adverse events were identified or reported, and the participant expressed the willingness to continue using the PR-ENDS device, they had the option to be enrolled in an extension of the trial. The first data point of PR-ENDS use in the behavior assessment was observed on February 22, 2022, and as of April 21, 2022, more than 200,000 individual puff data had been collected. The duration of the actual use observation of the PR-ENDS device in this paper is therefore considered 2 months.

### Materials and Measures

During the actual use trial, participants were asked to use the PR-ENDS device as their primary source of nicotine with selected e-liquids. The number and duration for each puff were directly recorded by PR-ENDS and uploaded to the app, and synchronized with the cloud. The nicotine emission per PR-ENDS puff was estimated based on recorded puff duration, e-liquid nicotine concentration, device power, and nicotine emission laboratory testing result. The detailed computational formula and assumptions applied in the calculation are listed in Appendix S1 in Multimedia Appendix 1 (also see Figure S1) [28,29].

### Statistical Analysis

Descriptive statistics, including mean (average), SD, and SE were calculated. The statistical significance is rather uninformative due to the large puff sample size (>200,000 puffs) [30]. Instead, *R*^2^ coefficient and Cohen *d* (for each categorical variable) were calculated to interpret the magnitude of effect size. Coefficient of variance was also calculated to estimate the variability of parameters such as daily puff number, puff duration, and daily nicotine consumption.

### Ethical Considerations

This observational study is on a commercially soft-launched product in the United Kingdom. Customers voluntarily participated and signed the product trial agreement with the distributor (Totally Wicked). The participants consented to have the puff behavior data recorded by PR-ENDS and used for market research, scientific research, and publications. No personally identifiable information was collected and available to the authors. Upon completing the product trial, participants received £30 (US $36) Totally Wicked store credit to redeem for their subsequent purchases.

## Results

### Participant Demographic and Nicotine History

Information about participants’ demographics and nicotine history collected from the baseline survey is presented in Table 1. Throughout the observation of the actual use of the PR-ENDS device, 58/61 participants provided the device ID, which allowed us to identify 200,411 individual puff data uploaded to the cloud and to associate with the actual use behavior recorded in real-time. As shown in Table 1, similar puff usage of the PR-ENDS device was observed between female and male users; young adults (ie, 18-25 years old; UK minimum legal age to smoke is 18) and “never smokers” typically recorded low usage of PR-ENDS, contributing only 4.6% (9304/200,411) and 13.8% (27,607/200,411), respectively, to the total puffs recorded. Of note, participants of age 36-55 with cigarette smoking history and 6-10 years of ENDS use were recognized as the most prevalent cohort in the user population.

**Table 1:** Summary of participants’ demographics and nicotine history (n=61) and PR-ENDS^a^ puff distribution (n=200,411).

Parameters		Participants, n (%)	PR-ENDS puffs, n (%)
**Sex**			
	Female	31 (50.82)	98,619 (49.21)
	Male	29 (47.54)	100,899 (50.35)
	Prefer not to say	1 (1.64)	893 (0.45)
**Age**			
	18-25	7 (11.48)	9304 (4.64)
	26-35	16 (26.23)	58,099 (28.99)
	36-55	33 (54.10)	92,327 (46.07)
	≥56	5 (8.20)	40,681 (20.30)
**With cigarette smoking history**			
	Yes	23 (37.70)	106,647 (53.21)
	No	7 (11.48)	27,607 (13.78)
	Did not respond	31 (50.82)	66,157 (33.01)
**Years of ENDS use**			
	6 months to 1 year	4 (6.56)	23,060 (11.51)
	1-5 years	26 (42.62)	51,306 (25.60)
	6-10 years	23 (37.70)	94,923 (47.36)
	>10 years	6 (9.84)	30,832 (15.38)
	Did not respond	2 (3.28)	290 (0.14)

^a^PR-ENDS: puff recording electronic nicotine delivery system.

### Device Power and Nicotine Concentration

As shown in Multimedia Appendix 2A, the high, medium, and low power of the PR-ENDS device contributed 46.43% (93,062/200,411), 37.43% (75,016/200,411), and 16.13% (32,333/200,401), respectively, to the total puffs, which indicated that all 3 power settings were sufficiently used (at least 30,000 puffs) by participants (Table S1 in Multimedia Appendix 1). About 60% of total recorded puffs (118,949/200,411, 59.35%; Table S2 in Multimedia Appendix 1) contained information on the nicotine concentration of the e-liquids used, and the distribution of puffs by nicotine concentration is presented in Multimedia Appendix 2B; 3 mg/mL (32,602/200,411, 16.26%) and 6 mg/mL (32,333/200,401, 16.13%) were recognized as the most prevalent nicotine concentrations for e-liquids used in the PR-ENDS device, followed by 11 mg/mL (14,480/200,401, 7.22%), 18 mg/mL (13,533/200,401, 6.75%), and 14 mg/mL (8695/200,401, 4.32%). Certain nicotine concentrations were of lower use than that from similar concentrations such as 1 mg/mL (compared with 3 mg/mL) and 10 mg/mL (compared with 11 mg/mL). This is likely due to the low availability of such e-liquid nicotine concentration in the UK market or the e-liquid preference of the population.

The PR-ENDS device was designed with 3 discrete power settings for the purpose of supplying different ranges of wattage (low: 7-9 W; medium: 9-11 W; and high: 11-13 W) to heat e-liquids. During the actual use session, the contribution to PR-ENDS total puffs was divided by combinations of different power settings and e-liquid nicotine concentrations (Table S3 in Multimedia Appendix 1). To simplify the presentation, e-liquid nicotine concentrations were categorized into 0 nicotine (0 mg/mL), low nicotine (≤6 mg/mL), medium nicotine (6-14 mg/mL), and high nicotine (≥14 mg/mL), with results presented in Table 2.

**Table 2:** PR-ENDS^a^ puff distribution (%) by the combinations of device power and nicotine concentration (n=118,947).

Nicotine concentration (device power)	Low power (7-9 W), n (%)	Medium power (9-11 W), n (%)	High power (11-13 W), n (%)
Zero nicotine (0 mg/mL)	4633 (3.90)	517 (0.43)	0 (0)
Low nicotine (≤6 mg/mL)	6464 (5.43)	22,933 (19.28)	36,108 (30.36)
Medium nicotine (6-14 mg/mL)	2955 (2.48)	12,361 (10.39)	3075 (2.59)
High nicotine (≥14 mg/mL)	4816 (4.05)	10,996 (9.24)	14,089 (11.84)

^a^PR-ENDS: puff recording electronic nicotine delivery system.

Across all 3 device powers, low nicotine e-liquid was the most prevalently used nicotine concentration. Such a result is consistent with the observation that 3 and 6 mg/mL were the most popularly used with PR-ENDS. Interestingly, for 0 nicotine e-liquid, the most prevalently used device power is low power. Much fewer puffs were generated using medium or high power with the 0 mg/mL e-liquid. For low and high nicotine e-liquids, the most prevalently used device power was high power, followed by medium and low power, whereas for medium nicotine e-liquids, medium power was the most prevalently used device power (ie, medium power and 11 mg/mL in Table S3 in Multimedia Appendix 1). The reason for the observed complex interactions between device power and nicotine concentration is unknown. However, a hypothesis to explain this phenomenon might be that some users may prefer certain power settings for a certain range of nicotine concentrations, such as a relatively high device power with low nicotine e-liquids or vice versa. Other users have opposite preferences such as a high device power with high nicotine e-liquids to reduce craving or a low device power with low/zero nicotine e-liquids to cut back nicotine use. What is worth noting here is that medium power showed a much higher PR-ENDS usage with medium nicotine e-liquids (compared with low and high power), which would lead to higher consumptions of nicotine in actual use. The implication of this finding will be further discussed in the following sections.

### Puff Duration Recorded by PR-ENDS

**Figure 1:**
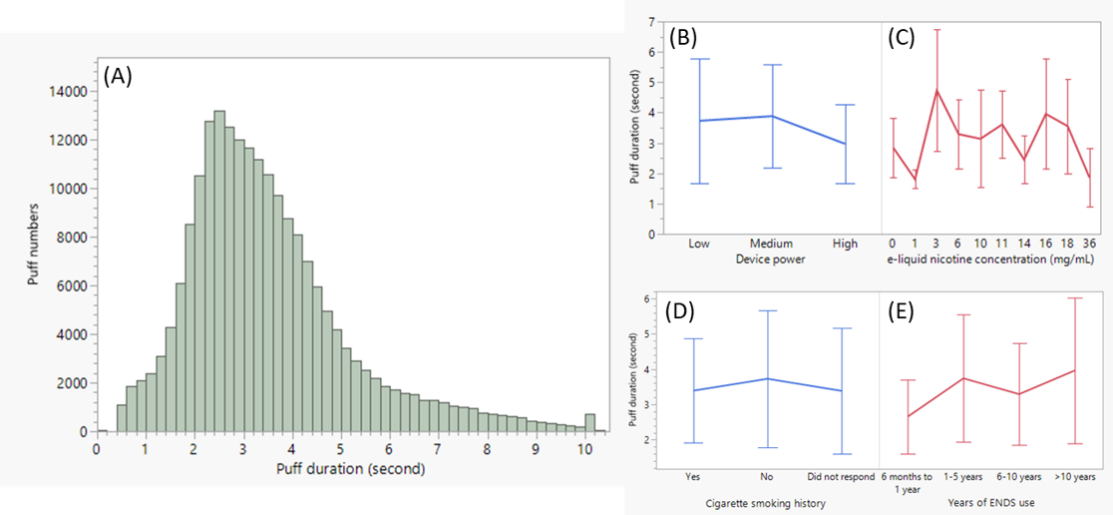
(A) The distribution of PR-ENDS puff duration. Comparison of puff duration by (B) device power, (C) e-liquid nicotine concentration, (D) cigarette smoking history, and (E) ENDS use history. The line represents the average value, and the error bar represents the SD.

As an observation platform, PR-ENDS enables naturalistic and noninvasive assessment of puff topography and puffing behaviors, where users can use the device freely with no interference from investigators. Based on 200,411 individual puff data, the distribution of PR-ENDS puff duration in actual use is displayed in Figure 1A. The puff duration distribution is slightly right skewed, with mean and median values of 3.44 and 3.10 seconds, respectively. The observed small tail in puff duration distribution (at 10 seconds) can be explained by the automatic power shutdown mechanism built in the PR-ENDS device, which means puffs longer than 10 seconds are not possible. A similar phenomenon has been identified from an equivalent power regulation model in previously published results [7]. It is noted that the observed average puff duration of 3.44 (SD 1.65) seconds is close to the puff duration (3 seconds) specified in [31] and [32], which has been widely recognized and applied in aerosol testing for harmful and potentially harmful constituents and cellular toxicology assessment for regulatory submissions.

### Effects of Different Factors on Puff Duration

During the actual use, various factors, including device, e-liquid, and user profile, would influence the puff duration. To assess the effect of different factors on PR-ENDS puffs, the measured puff durations were compared by device power, e-liquid nicotine concentration, and nicotine use history (cigarette and ENDS). Given the large puff sample size (>200,000 puffs), the statistical difference in puff duration identified by variable comparisons such as ANOVA is uninformative (ie, showing the significant difference with *P*<.001 regardless of the selected variables; data not shown). Instead, *R*^2^ coefficient and Cohen *d* (for categorical variable) are better suited for interpreting the magnitude of effect size in statistical analysis.

Based on the *R*^2^ coefficients in Table 3, device power, nicotine concentration, and nicotine use history (cigarette and ENDS) all only have small effects (*R*^2^<0.10) on PR-ENDS puff duration. As shown in Figure 1B, low, medium, and high power yielded puff durations with comparable mean values, although it is recognized in Table 3 that the difference of puff duration between medium and high power is moderately significant (Cohen *d*=0.573). Based on the comparisons shown in Figure 1C-E, e-liquid nicotine concentration, cigarette smoking history, and years of ENDS use seemed to have no obvious effects on puff duration (*R*^2^=0.005-0.060). However, more years of ENDS use is moderately associated with higher puff durations, with the results shown in the differences of puff durations between >10 years and 6 months to 1 year, and between 1-5 years and 6 months to 1 year (Figure 1E).

**Table 3:** Summary of effect size (R2 coefficient and Cohen d) on puff duration by device power, e-liquid nicotine concentration, and nicotine (cigarette and ENDS^a^) use history.

Factors			*R*^2^ coefficient		Cohen *d*^b^
Device power			0.069		0.573^c^
Nicotine concentration			0.060		N/A^d^
Cigarette smoking history			0.005		N/A
**Years of ENDS use**			0.054		
	>10 years versus 6 months to 1 year			0.814	
	1-5 years versus 6 months to 1 year			0.676	

^a^ENDS: electronic nicotine delivery system.

^b^Only medium to large effect size (Cohen *d*≥0.5) is presented.

^c^Medium power versus high power.

^d^“N/A” denotes “not applicable” or small effect size (Cohen *d* <0.5).

### PR-ENDS–Based Nicotine Emission

In our recent study [25], the feasibility of PR-ENDS in estimating user’s nicotine consumption in a habitual environment has been demonstrated. Specifically, with recorded puff duration, e-liquid nicotine concentration, device power, and laboratory-tested nicotine emission in place, the nicotine emission (per puff) and nicotine consumption during PR-ENDS actual use can be approximated, which is suitably correlated with the consumed nicotine level derived from e-liquid weight loss and pharmacokinetic parameters (ie, area under the pharmacokinetic nicotine concentration-time curve). The detailed equations and assumptions applied in calculations for PR-ENDS–based nicotine emission are listed in Appendix S1 in Multimedia Appendix 1.

**Figure 2:**
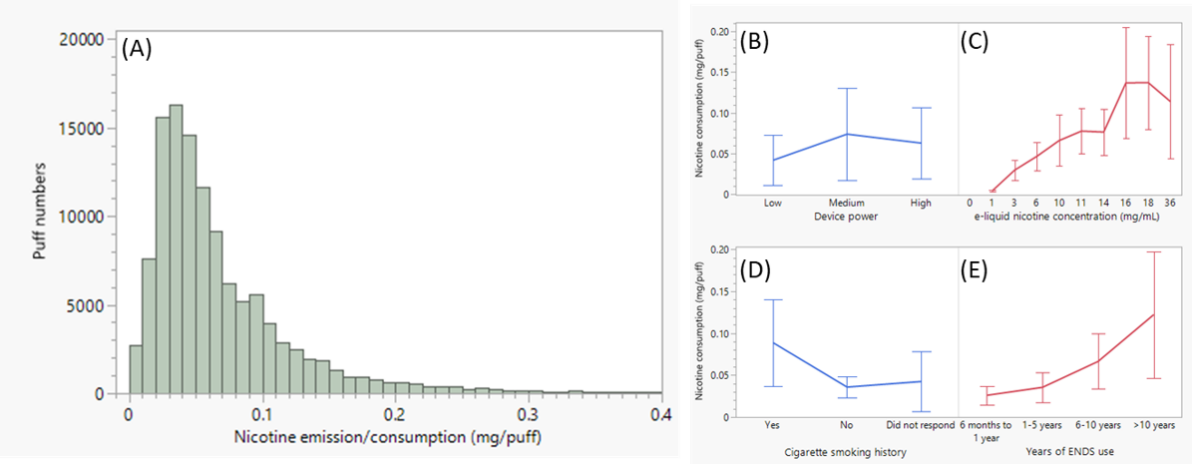
(A) The distribution of PR-ENDS nicotine emission. Comparison of nicotine emission (mg/puff) by (B) device power, (C) e-liquid nicotine concentration, (D) cigarette smoking history, and (E) ENDS use history. The line represents average value, and the error bar represents SD. ENDS: electronic nicotine delivery system.

Based on 113,797 puff data collected on PR-ENDS with a record of nonzero nicotine concentrations (Table S2 in Multimedia Appendix 1), the distribution of PR-ENDS–derived nicotine emission (per puff) is displayed in Figure 2A. The distribution is right skewed with mean and median values of 0.0648 and 0.0508 mg/puff, respectively. It is known from previously published data [33] that a heated tobacco product (ie, IQOS) and a reference combustible cigarette (ie, 3R4F) emit about 0.129 and around 0.174 mg/puff of nicotine under the laboratory test condition [32]. Although it is challenging to directly compare the nicotine emissions between PR-ENDS and the 2 aforementioned nicotine products, preliminary tests on PR-ENDS aerosols have shown that the device yields a nicotine emission of about 0.0952 mg/puff (Table S4 in Multimedia Appendix 1) when used with 12 mg/mL e-liquid and high power, and operated with the Coresta puff regime (55 mL/3 seconds/30 seconds) [32]. This is the same puff regime tested for IQOS and combustible cigarettes. Considering that lower device powers (low and medium power) and lower nicotine concentrations of e-liquids (ie, 3 and 6 mg/mL) were prevalently used during actual use, the currently observed PR-ENDS nicotine emissions are deemed reasonable. They are lower than the laboratory testing result, and are generally lower than that from commonly used nicotine products such as IQOS and combustible cigarettes.

### Effects of Different Factors on Nicotine Emissions

To assess the effect of different factors on nicotine emissions during PR-ENDS actual use, the calculated values were compared by device power, e-liquid nicotine concentration, and nicotine use history (cigarette and ENDS) accordingly. Based on *R*^2^ coefficient in Table 4, the effect of device power on nicotine emission is considered small (*R*^2^=0.042). Albeit, interestingly, as shown in Figure 2B, medium power yielded the highest average nicotine emission per puff compared with high and low power, and the difference in nicotine emission is moderately significant between medium and low power (Cohen *d*>0.5), but not between high and low power (Cohen *d* < 0.5). Such observations indicated that users preferred applying medium power instead of low or (even) high power for a higher nicotine emission within a single PR-ENDS puff. This can be ascribed to the complex interaction effect of combining different device power and e-liquid nicotine concentrations during the actual use of ENDS [34], and the previous discussion has suggested that a much higher contribution of using medium nicotine e-liquids was preferably associated with medium device power (Table 2). As a consequence, medium power yielded the highest nicotine emission compared with low and high power.

**Table 4:** Summary of effect size (R2 coefficient and Cohen d) on nicotine emission per puff by device power, e-liquid nicotine concentration, and nicotine (cigarette and/or ENDS^a^) use history

Factors		*R*^2^ coefficient	Cohen *d*^b^
Device power		0.042	0.666^c^
Nicotine concentration		0.422	N/A^d^
**Cigarette smoking history**		0.246	
	Yes versus no		1.228
	Yes versus did not respond		1.078
**Years of ENDS use**		0.351	
	>10 years versus 6 months to 1 year		2.432
	>10 years versus 1-5 years		2.190
	>10 years versus 6-10 years		1.410
	6-10 years versus 6 months to 1 year		1.022
	6-10 years versus 1-5 years		0.781

^a^ENDS: electronic nicotine delivery system.

^b^Only medium to large effect size (Cohen *d*≥0.5) is presented.

^c^Medium power versus low power.

^d^“N/A” denotes “not applicable” or small effect size (Cohen *d*<0.5).

In contrast to device power, which only generated a small effect on nicotine emission, e-liquid nicotine concentration rendered a relatively large effect (*R*^2^=0.422), followed by years of ENDS use (*R*^2^=0.351) and cigarette smoking history (*R*^2^=0.246). Coherent with high *R*^2^ coefficients, Figure 2C showed that a higher nicotine emission per puff is strongly correlated with a higher e-liquid nicotine concentration, especially in the range from 0 to 16 mg/mL. For nicotine use history, based on comparisons shown in Figure 2D,E, both cigarette smoking and years of ENDS use have moderate correlation effects with nicotine emission per puff. Such a result is not surprising, given the fact that experienced e-cigarette users tend to puff more intensively [11] with higher nicotine doses [35]. As a result, PR-ENDS users with a cigarette smoking history and more years of ENDS use tended to consume more nicotine per puff with large Cohen *d* values (Table 4).

### Individual Puffing Behaviors Observed Over Time

The sections above have demonstrated that the PR-ENDS device is capable of measuring actual use puffing behavior as well as their correlations with various use factors in real-world settings. Furthermore, when the device is securely connected to device app with a smartphone or a computer via Bluetooth, the puff data can be uploaded to the cloud for real-time monitoring of product use behavior. Such a feature not only empowers individuals with the awareness to help them quit or cut back their nicotine use, but also provides an effective observation platform for assessing individual and group puffing behaviors and understanding any potential use trends or patterns as proactive postmarket surveillance. With the real-time puff data of 58 users collected for 2 months (from February 22 to April 21, 2022), the daily puff numbers, puff durations, and daily nicotine consumptions for each user can be calculated and the statistics of actual use puffing behavior parameters are summarized in Table S5 in Multimedia Appendix 1.

**Figure 3:**
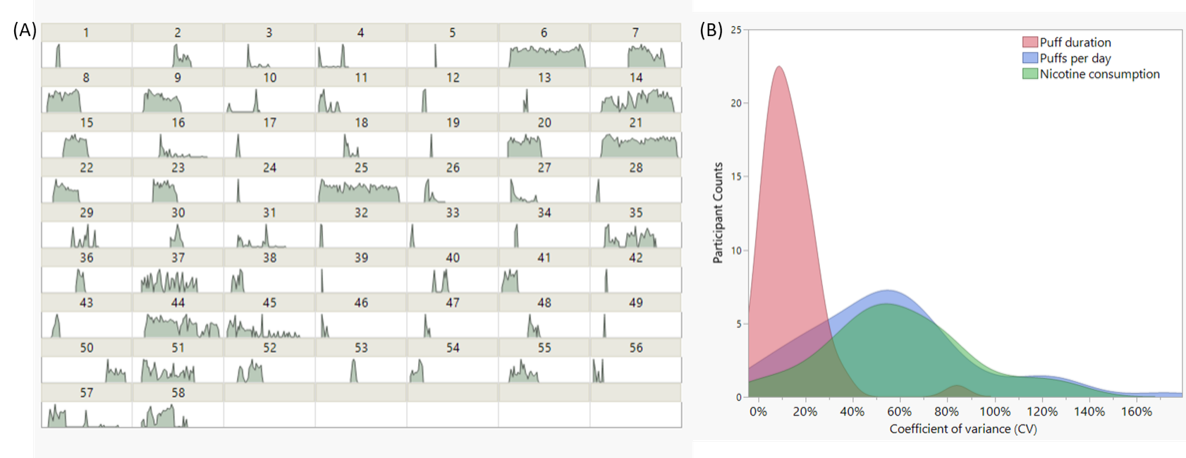
(A) Daily histogram (normalized) representation of puff numbers for each participant (1-58). (B) Participant distributions of their coefficient of variance in daily puff duration, puffs per day, and daily nicotine consumptions over the active period.

The real-time PR-ENDS use (in daily puff numbers per user) is presented in Figure 3A as the normalized plot histogram of puff number versus date. Notable differences in use patterns can be seen between different individuals. For example, certain participants (ie, participants 6, 21, 25) continuously used PR-ENDS for more than 50 days with relatively stable use intensity (daily puff numbers), while others tended to use the device somewhat sporadically (3, 4, 16, 46-49, etc.), with following days inactive in product use. Some participants (1-7, 25-28, etc.) used PR-ENDS with less than 250 puffs per day during the actual use, yet others used PR-ENDS more intensively, with more than 400 puffs (22, 23, 43, 54, etc.) recorded per day. Based on the summarized data in Table S5 in Multimedia Appendix 1, the puff durations of participants vary from 0.90 to 6.87 seconds; the daily puff numbers vary from 5 to more than 400; and the daily nicotine consumptions vary from 0.08 to 36.24 mg.

Besides the diverse PR-ENDS use patterns in different participants, substantial variabilities of puffing behaviors within the same user profile over time can be found. Figure 3B shows the distribution of coefficient of variance for participants in daily puff numbers, daily average puff duration, and daily nicotine consumptions over their own active period. Most participants had large variances (0%-160%) in daily puff numbers and daily nicotine consumptions. However, their daily average puff durations were much less variable, with most participants’ coefficient of variances located in the range of 0%-40%. The significant variability of puff number and nicotine consumption within each individual participant represented the actual use situation in real-world settings. It is significant that users’ puffing behavior, aside from puff duration, did not present a consistent use format, but rather evolved dynamically over time. For example, users had higher puff numbers and nicotine consumptions on certain days yet had lower puff numbers and nicotine consumptions on other days. However, most participants’ daily puff durations during actual use were relatively stable, and no significant variance was identified over the observation period. Such results should prompt further investigations on the real-time e-cigarette puffing behavior assessments with a larger sample size and a longer study duration in the future.

### Populational Puffing Behaviors Observed Over Time

From the perspective of group behavior assessment, longitudinal observations of PR-ENDS puffing behaviors over time (ie, 3 weeks or longer) should be treated as strong indicators to interpret the product-specific nicotine addiction potential and abuse liability [36,37]. Specifically, when the observed participant group is being treated as a cohort, their first recorded day of using PR-ENDS can be considered as day 1 in the longitudinal observation. Thus, puffs per day can be calculated by taking puff numbers from active users in each day into account. Puff duration per day can be calculated by averaging the puff duration of the active users in each day. Nicotine consumption per day can be obtained by calculating accumulated puffs with nicotine consumption in each puff of active users in each day. The detailed calculation rationale is listed in Appendix S2 in Multimedia Appendix 1.

**Figure 4:**
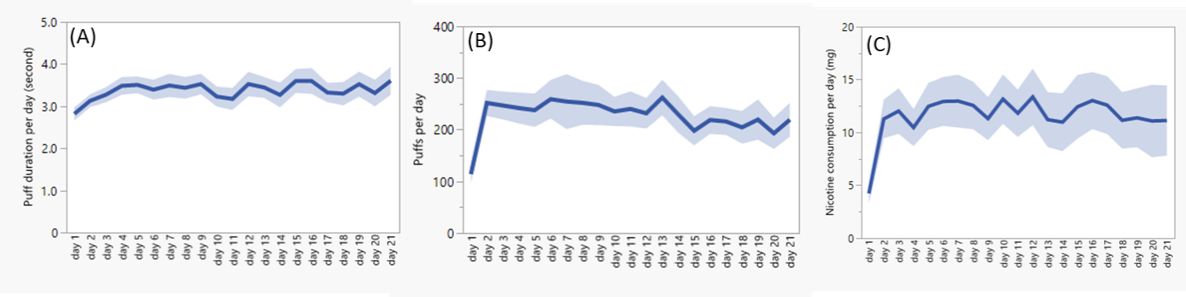
Observation of (A) puffs per day, (B) puff duration per day, (C) nicotine consumption per day of the user group during the actual use of PR-ENDS over 3 weeks. The line represents the average, and the band represents the SE. PR-ENDS: puff recording electronic nicotine delivery system.

For puffs per day, as shown in Figure 4A, the participant group initiated the actual use of the PR-ENDS device with about 120 puffs on the first day (day 1), and the group quickly adapted to “normal usage” of about 250 puffs per day after 1-2 days (days 2-3). The puffs per day value then stabilized over time until the end of the third week. Over the time span of 3 weeks, the participant cohort consistently used the PR-ENDS device with no observable increase in puffs per day over time. This finding is consistent with the examination of daily puff numbers for each individual that no obvious ramp-up trends were identified in Figure 3A. Figure S2 in Multimedia Appendix 1 further assessed the robustness of this observation, with a similar trend identified for users who used PR-ENDS for 1, 2, 4, 5, 6, and 7 weeks. In all figures, PR-ENDS use pattern showed an initial low puff number on the first day, followed by a quick increase and plateauing of puffs per day due to adaptation to habitual use.

For puff duration per day, as shown in Figure 4B, the observed group initiated the use with an average puff duration of about 2.8 seconds on day 1 and the value gradually increased and plateaued to about 3.5 seconds after about 5-7 days. This puff duration trend was consistently maintained until the end of the third week (21 days). The stabilized puff duration was found to be consistent with the puff duration (mean 3.44 seconds, SD 1.65 seconds) reported in Figure 1A. The low intraindividual variability in puff durations in Figure 3B also validated the consistent puff durations. This further indicates that after acclimatization to the use of PR-ENDS, participants consistently used the device with limited abuse tendency and with no significant increase in puff durations after 1 week and beyond.

Lastly, for nicotine consumption per day of the cohort group, as shown in Figure 4C, it is observed that participants started using PR-ENDS with an average daily nicotine consumption of about 4.2 mg on day 1. The user group then quickly adapted to “normal usage,” with nicotine consumption per day increasing and plateauing at about 12 mg/day after 1-2 days. Nicotine consumption per day then stabilized until the end of the third week. The trend of nicotine consumption over time observed was almost the same as puffs per day, which indicates either that user did not change e-liquid nicotine concentration over the observation period or that the user did not update their app record after such a change.

## Discussion

### Principal Findings

In this report, actual use behavior assessments of a novel PR-ENDS device during a 2-month long observation session were discussed. It was found that the PR-ENDS device was primarily consumed by existing nicotine product users who are well past young adulthood, which is likely due to the design feature of PR-ENDS as a complicated open e-cigarette system that entails smoking or vaping experience. It is expected that nicotine naïve users are not primarily interested in using this device. As an ENDS product that requires e-liquid refill and wattage adjustment (3 discrete power settings: low, medium, and high), a diverse range of e-liquid nicotine concentrations and a complex interactive effect between e-liquid nicotine concentrations and device powers were recognized based on information collected from PR-ENDS. For example, high power was recognized as the most prevalently used power setting when the device was combined with low (≤6 mg/mL) and high (≥14 mg/mL) nicotine e-liquids; medium power of the device contributed much more puffs with medium (6-14 mg/mL) nicotine e-liquids, whereas low power is predominantly used for 0 nicotine e-liquid (0 mg/mL). Such observation highlighted the importance of being able to provide a wide range of device powers and e-liquid nicotine concentrations during e-cigarette actual use for reducing nicotine craving and smoking transition. In fact, previous clinical trials have clearly proven that open refillable e-cigarettes that enable free choices of e-liquids led to more significant smoking abstinence rates compared with self-abstinence and nicotine-replacement groups [38,39].

The PR-ENDS device–collected information showed a reasonable distribution of puff duration (mean 3.44 seconds, SD 1.65 seconds) based on >200,000 individual puff data. The observed puff duration during actual use is very close to the value proposed in aerosol testing protocols [31,32]. The value aligned well with the puff duration data published from other e-cigarette use behavior studies, where puffs were found to last from 2 to 4 seconds during the actual use [7,8,10,11,40-43]. The statistical significance can consistently be seen when PR-ENDS puff durations were compared under different device power, nicotine concentration, or nicotine history (data not shown). However, calculations on the effect size (*R*^2^ coefficient and Cohen *d*) unveiled that none of the aforesaid factors yielded a significant change in puff duration during actual use.

Considering that the puff topography and PR-ENDS–specific information in estimating nicotine consumption (ie, device power, e-liquid) was collected in situ, it is viable to evaluate the PR-ENDS device–based nicotine emission estimates, as well as to assess the implications on nicotine use by comparing nicotine emissions under different factors. For example, it was observed that medium power was associated with the highest average nicotine emission per puff compared with low or high power. This is probably due to the fact that medium power was much more frequently used with medium (6-14 mg/mL) and high (≥14 mg/mL) nicotine e-liquids and that higher nicotine concentrations are prone to yield higher nicotine emissions in product use [28]. What is interesting about the current finding is that the change in nicotine emission per puff is not directly proportional to the increase of the PR-ENDS device power from low to medium to high. Instead, the free selection of e-liquid nicotine concentrations rendered the actual use inevitably more complex (ie, medium power was associated with the highest nicotine emissions instead of high power). In addition, higher nicotine concentrations, cigarette smoking history, and more years of ENDS use all led to higher nicotine emission per puff with relatively significant effect size, although the associated puff durations are relatively comparable and with a small effect size. As identified from previous research, e-cigarette users would attempt compensatory puffing patterns and nicotine self-titrations [8], with puff number and puff durations being lower while liquid and nicotine consumption being higher when they used e-cigarettes with a higher power setting. However, in the current actual use observation, we found that the compensatory puff pattern is not significant (small effect on puff duration) while nicotine emission was strongly correlated with various factors (large effect on nicotine emission). Such a contrasting result brought further contextualization to the identified confounding effects here, as the selection of device power, e-liquid nicotine concentration, puff topography, and nicotine consumptions are all interrelated to each other and are affected by the puffing behavior and nicotine history during the actual use. It would be reasonable to contend from these findings that choices in nicotine concentration and device power settings are important influences on the behavior of e-cigarette users. The more “subconscious” influence of puff duration shows much lower variability, meaning that users’ self-titration of nicotine consumption is likely to be a conscious choice.

With puff data recorded in real-time, we further considered the time course of PR-ENDS puffing behavior at both individual and population levels. When the puff data are viewed by each user profile over the period of actual use, different product use patterns in daily puff numbers can be recognized such that some participants had a consistent trend in puffs per day with PR-ENDS, while others chose to use the device sporadically without continuity of use over time. Further, the calculation of the coefficient of variance from puffs per day and nicotine consumption for the same PR-ENDS user showed a large variability over their own active period. These observations, taken together, highlighted the considerable unpredictability in both inter and intraindividual actual use of puffing behaviors and emphasized the importance of discovering puffing behavior patterns at the individual level with real-time feedback.

By contrast, the time course of the entire PR-ENDS cohort consistently showed a quick adaptation to device use followed by a consistent use pattern. As presented in Figure 4A-C, the participant group initiated the use of PR-ENDS with about 120 puffs and 2.8-second puff duration on day 1, and then quickly adapted to normal usage of 250 puffs and 3.5-second puff duration in 1-2 days and 5-7 days, respectively. After 1 week, the daily puff number and puff duration of the user group stabilized and plateaued until the end of day 21. The initial increase in daily puff numbers and puff durations observed for PR-ENDS is consistent with the findings of a recently published study [6], where the authors concluded that users tended to prolong their puffs using a 10-W ENDS device filled with 6 mg/mL e-liquid over 5 consecutive days (the study period). Considering the similarity of the device power and e-liquid nicotine concentration between the 2 investigated ENDS devices, the identification of the same trend in increasing the use of ENDS is not surprising. However, the observation duration in this study was at least 21 days, which is much longer than 5 days in the previous study, allowing us to identify trends over a relatively long term. This included the consistent use pattern of puff number and puff duration after acclimatization to the use of PR-ENDS following 1 week of use.

The observed trend of the PR-ENDS device–estimated daily nicotine consumption rendered a similar use pattern over time as the daily puff number in the user group. The users’ daily nicotine consumption was observed with an initial increase step in 1-2 days followed by consistent use for at least 3 weeks or 21 days. The eventually stabilized daily nicotine consumption was found to be around 12 mg/day. A direct comparison of daily nicotine consumption between PR-ENDS and other nicotine products is challenging. However, based on a previous study conducted on the daily intake of nicotine from cigarette smoking [44] with an average nicotine consumption of 37.6 mg, the nicotine consumption calculated from PR-ENDS is only about 30% of the nicotine intake from smoking per day. It should be noted that the nicotine consumption in this observation is rather theoretical and only based on laboratory testing results, while the previous study on nicotine intake was conducted with blood specimen analysis. Further studies are warranted to investigate the overall nicotine consumption from PR-ENDS users (including smoking and other nicotine products) during actual use.

We acknowledge that the actual use behavior assessment has a few limitations. First, because the assessment was conducted in a real-world condition, the participants were not closely monitored but only traced by the follow-up survey. As a result, 3/61 participants did not provide their PR-ENDS device ID, which did not allow the association with their actual use behavior. However, it is believed that the reported puff data of 58 participants with 200,411 puffs are sufficient to depict the actual use behavior of the observed PR-ENDS population throughout the period of 2 months. Second, the e-liquid information (ie, nicotine concentration) collected from the PR-ENDS device was self-reported by users and was used to estimate the nicotine consumption per puff and per day. The calculation was theoretical and based on certain assumptions that have not been experimentally verified, although a similar example in nicotine consumption estimation has been clinically validated [25] and is recognized in this report as the close proxy of nicotine use in the real world. Third, the ability to draw concrete behavioral conclusions based on the duration and sample size of the current observational assessment is still limited. Importantly, although test participants were asked to use PR-ENDS as their primary source of inhaled nicotine for the duration of the observation period, it is likely that some users were not using PR-ENDS exclusively. Future prospective studies with longer term [45] and bigger groups [38] are expected to further improve the study design and conclusions.

Some key strengths of the actual use behavior assessment are as follows: (1) This is the first observational behavior assessment conducted in real-world conditions that systematically examined the puff topography and puffing behavior at both individual and population levels. (2) For the first time, we identified the complex interactions between device power and e-liquid nicotine concentrations in e-cigarette actual use and their effects on puff topography and use behaviors. (3) The real-time observations on PR-ENDS actual use revealed that a significant variability of puffing behaviors exists between different users and within the same individual user over time. A quick adaptation pattern (an increase of puff number and puff duration followed by stabilized product use for at least 3 weeks) was observed for the first time when the cohort was assessed as a whole.

### Conclusions

In summary, a real-time, actual use behavior assessment was conducted to investigate PR-ENDS–specific puff topography and puffing behaviors at both individual and population levels. It was observed that middle-aged adults with nicotine use history represented the major user profile, and a wide range of device power and e-liquid nicotine concentrations was applied during PR-ENDS actual use. Various puff topography and puffing behavior parameters (ie, puff duration, puff number, estimated nicotine consumption) were assessed with the identification of contributing factors from device, e-liquid, and user nicotine history. The real-time observations of PR-ENDS puffing behavior further revealed the intra- and interindividual behavior variabilities, as well as a quick adaptation pattern followed by stabilized product use for at least 3 weeks.

## Appendix

Multimedia Appendix 1 Supporting information.

Multimedia Appendix 2 PR-ENDS puff distribution by (A) device power (B) e-liquid nicotine concentration. PR-ENDS: puff recording electronic nicotine delivery system.

## Abbreviations

e-cigarette: electronic cigarette
PG: propylene glycol
PR-ENDS: puff recording electronic nicotine delivery system
VG: vegetable glycerin

## Data Availability

The data sets generated during or analyzed during this study are not publicly available due to all PR-ENDS proprietary data and summary currently undergoing the FDA evaluation process but are available from the corresponding authors on reasonable request.
