# Supplementary material for: Actual Use Behavior Assessment of a Novel Puff Recording Electronic Nicotine Delivery System: Observation Study
Source: JMIR Form Res. 2023 Feb 8;7:e43175. doi: 10.2196/43175 (PMC9947749; doi:10.2196/43175)
Supplement: Multimedia Appendix 1 [file formative_v7i1e43175_app1.docx]

Supporting Information

Actual Use Behavior Assessment of a Novel Puff Recording Electronic Nicotine Delivery System: An Observation Study

**Xiang Gao ^1, *^, Liam Humberstone ^2^_,_ Yatao Liu ^1, *^**

**^1^ Scientific Horizons Consulting, Irvine, CA, USA 92617**

**^2^ Totally Wicked Limited, Stancliffe Street, Blackburn, Lancashire, BB2 2QR**

* Correspondence:

[xiang.gao@scientifichorizonsconsulting.com](mailto:xiang.gao@scientifichorizonsconsulting.com)

[yatao.liu@scientifichorizonsconsulting.com](mailto:yatao.liu@scientifichorizonsconsulting.com)

**Declaration of competing interests:** Authors declare no conflicts of interest.

Table S1 PR-ENDS puff distribution by power settings (n = 200,411)

| **Device power** | **Number of puffs (n)** | **Percentage of the total puffs (%)** |
| --- | --- | --- |
| High | 93,062 | 46.4% |
| Medium | 75,016 | 37.4% |
| Low | 32,333 | 16.1% |

Table S2 PR-ENDS puff distribution by e-liquid nicotine concentrations (n = 200,411)

| **E-liquid nicotine concentration** | **Number of puffs (n)** | **Percentage of the total puffs (%)** |
| --- | --- | --- |
| 0 mg/ml | 5,150 | 2.6% |
| 1 mg/ml | 570 | 0.3% |
| 3 mg/ml | 32,602 | 16.3% |
| 6 mg/ml | 32,333 | 16.1% |
| 10 mg/ml | 3,913 | 2.0% |
| 11 mg/ml | 14,480 | 7.2% |
| 14 mg/ml | 8,695 | 4.3% |
| 16 mg/ml | 4,555 | 2.3% |
| 18 mg/ml | 13,533 | 6.8% |
| 36 mg/ml | 3,118 | 1.6% |
| Blank* | 81,462 | 40.6% |

* Blank represents no information regarding the e-liquid nicotine concentration was provided by the PR-ENDS user.

Table S3 PR-ENDS puff distribution (%) by the combinations of device power and nicotine concentration

| **Nicotine concentration**  **Device power** | Low power  (7 - 9 watts) | Medium power  (9 - 11 watts) | High power  (11 - 13 watts) |
| --- | --- | --- | --- |
| 0 mg/ml | 3.9% | 0.4% | 0.0% |
| 1 mg/ml | 0.0% | 0.0% | 0.5% |
| 3 mg/ml | 3.6% | 13.0% | 10.8% |
| 6 mg/ml | 1.9% | 6.2% | 19.1% |
| 10 mg/ml | 0.5% | 1.1% | 1.8% |
| 11 mg/ml | 2.0% | 9.3% | 0.8% |
| 14 mg/ml | 1.1% | 1.7% | 4.6% |
| 16 mg/ml | 0.5% | 1.6% | 1.7% |
| 18 mg/ml | 1.1% | 5.9% | 4.4% |
| 36 mg/ml | 1.4% | 0.1% | 1.2% |

Appendix S1 Calculation Rationale for Nicotine Emission of PR-ENDS During Actual Use

Based on laboratory testing results shown in Table S4, it has been concluded that the nicotine emission (per puff) from PR-ENDS increases with the change of device power from Low to Medium to High power as well as from low nicotine concentration (3 mg/ml) to high nicotine concentration (12 mg/ml).

Table S4 Summary of nicotine emission (average) per puff for PR-ENDS

| **Device power** | **Puff duration*** | **Nicotine concentration of e-liquid (mg/ml)** | **Average nicotine emission (mg/puff)** |
| --- | --- | --- | --- |
| High | 3 second | 3 | 0.0180 |
|  | 3 second | 12 | 0.0952 |
| Medium | 3 second | 3 | 0.0144 |
|  | 3 second | 12 | 0.0762 |
| Low | 3 second | 3 | 0.0111 |
|  | 3 second | 12 | 0.0406 |

*The puff duration from laboratory testing was set as Coresta recommendation method (CRM) with 55ml volume, 3 second puff duration, and 30 second puff intervals.

Two assumptions were applied during the estimation of nicotine emission in PR-ENDS actual use:

1. The nicotine emission is linearly associated with the measured puff duration.

This is supported by previous studies (Hensel et al., 2021; Talih et al., 2015) that the puff flow rate alone does not significantly impact the aerosol emission and nicotine yield, and puff duration data is sufficient to represent the amount of nicotine generated from e-cigarettes.

1. The nicotine emission is linearly associated with the e-liquid nicotine concentration.

This is supported by the regression plots shown in Figure S2, where the plots must go through the origin point (0 mg/ml). A nearly perfect linear relationship (R^2^ > 0.99) between nicotine concentration (3 mg/ml and12 mg/ml) and nicotine emission exits across Low, Medium, and High device power, which indicates that the nicotine emission is linearly associated with the e-liquid nicotine concentration.

As such, nicotine emission per puff from PR-ENDS use can be estimated using the equation (1).

$$Nicotine emission per puff = \frac{{Nicotine emission}_{\mathrm{Testing}}}{{Puff druation}_{\mathrm{Testing}} \times{E-liquid nicotine concentration}_{\mathrm{Testing}}}$$

$\times{E-liquid nicotine concentration}_{PR-ENDS} \times{Puff duration}_{PR-ENDS}$ (1)

Specifically, nicotine emission_Testing_ data was provided in Table S1; puff duration_Testing_ and e-liquid nicotine concentration_Testing_ were set as 3 second and 3 mg/ml and 12 mg/ml, respectively. The data of e-liquid nicotine concentration_PR-ENDS_ and puff duration_PR-ENDS_ was directly downloadable from PR-ENDS app and cloud.


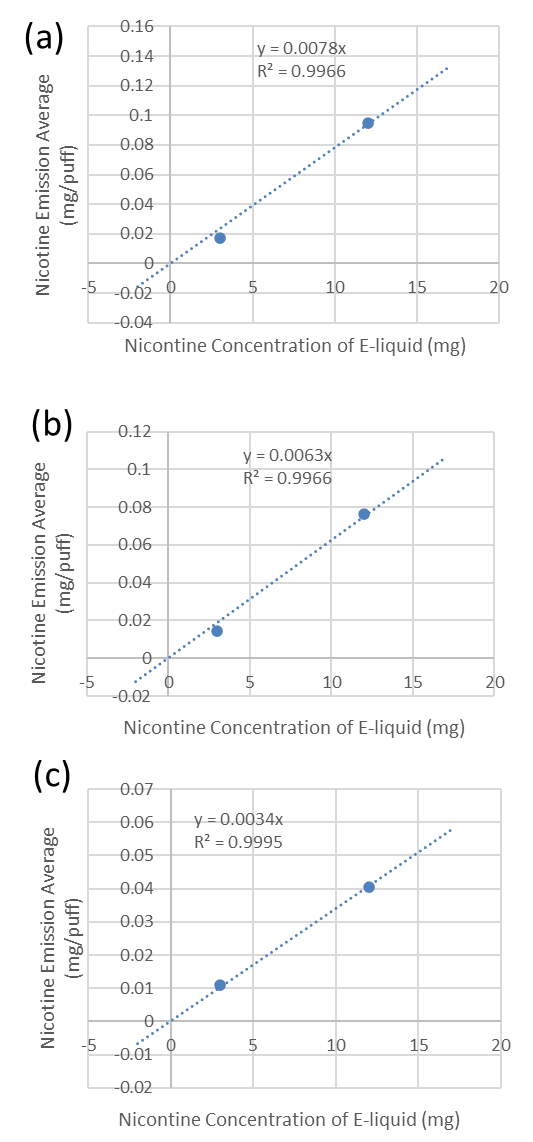


Figure S1 Linear regression plotting between nicotine emission per puff and e-liquid nicotine concentrations among (a) High, (b) Medium, and (c) Low device power.

Table S5 Summary of actual use puffing behavior characteristics over time by participants (N = 58).

|  | Daily puff duration (second) | | Daily puff numbers | | Daily nicotine consumption (mg) | |
| --- | --- | --- | --- | --- | --- | --- |
| Participant | Mean (SD^a^) | CV^b^ | Mean (SD) | CV | Mean (SD) | CV |
| 1 | 1.87 (0.06) | 3% | 96.67 (60.80) | 63% | 3.53 (2.49) | 70% |
| 2 | 3.00 (0.72) | 24% | 184.77 (102.55) | 56% | 6.68 (3.76) | 56% |
| 3 | 1.73 (0.43) | 25% | 54.09 (93.36) | 173% | /^c^ | / |
| 4 | 1.85 (1.55) | 84% | 67.90 (87.53) | 129% | 2.33 (3.00) | 129% |
| 5 | 1.52 (0.00) | 0% | 5.00 (00.00) | 0% | / | / |
| 6 | 3.80 (0.23) | 6% | 189.08 (31.62) | 17% | 9.60 (4.38) | 46% |
| 7 | 1.84 (0.26) | 14% | 157.81 (50.71) | 32% | 16.15 (7.42) | 46% |
| 8 | 2.54 (0.19) | 7% | 217.46 (53.61) | 25% | 23.63 (9.45) | 40% |
| 9 | 2.55 (0.48) | 19% | 364.15 (106.43) | 29% | / | / |
| 10 | 2.90 (1.01) | 35% | 42.75 (49.48) | 116% | 1.14 (1.19) | 104% |
| 11 | 2.16 (0.39) | 18% | 124.27 (79.16) | 64% | 4.27 (2.65) | 62% |
| 12 | 1.96 (0.14) | 7% | 110.33 (64.77) | 59% | 2.82 (1.69) | 60% |
| 13 | 4.53 (0.71) | 16% | 42.67 (26.56) | 62% | / | / |
| 14 | 3.87 (0.16) | 4% | 227.46 (97.40) | 43% | 12.54 (5.32) | 42% |
| 15 | 2.42 (0.25) | 10% | 354.33 (108.50) | 31% | 11.66 (4.97) | 43% |
| 16 | 3.65 (0.71) | 19% | 43.84 (55.05) | 126% | 2.75 (3.60) | 131% |
| 17 | 4.68 (0.40) | 9% | 115.67 (86.27) | 75% | / | / |
| 18 | 2.34 (0.57) | 24% | 14.60 (14.00) | 96% | 0.50 (0.49) | 99% |
| 19 | 1.83 (0.00) | 0% | 8.00 (00.00) | 0% | 0.08 (0.00) | 0% |
| 20 | 2.45 (0.19) | 8% | 334.46 (64.11) | 19% | 24.77 (6.29) | 25% |
| 21 | 3.58 (0.24) | 7% | 235.94 (50.76) | 22% | 18.78 (5.17) | 28% |
| 22 | 4.86 (0.61) | 12% | 412.68 (132.93) | 32% | 13.54 (4.60) | 34% |
| 23 | 2.36 (0.15) | 6% | 434.12 (89.15) | 21% | / | / |
| 24 | 3.29 (0.03) | 1% | 27.50 (22.50) | 82% | 1.71 (1.41) | 82% |
| 25 | 4.40 (0.31) | 7% | 214.04 (42.03) | 20% | 36.24 (6.81) | 19% |
| 26 | 5.50 (1.05) | 19% | 163.00 (156.93) | 96% | 3.73 (3.02) | 81% |
| 27 | 4.38 (0.98) | 22% | 22.71 (26.69) | 118% | 2.13 (2.55) | 120% |
| 28 | 1.51 (0.12) | 8% | 200.50 (112.50) | 56% | / | / |
| 29 | 1.89 (0.43) | 23% | 185.60 (133.09) | 72% | 0.67 (0.29) | 44% |
| 30 | 6.19 (0.45) | 7% | 357.33 (182.07) | 51% | 11.39 (8.37) | 73% |
| 31 | 3.61 (0.91) | 25% | 109.52 (139.03) | 127% | 1.91 (2.15) | 113% |
| 32 | 2.16 (0.01) | 1% | 290.50 (03.50) | 1% | 28.29 (0.87) | 3% |
| 33 | 2.64 (0.28) | 11% | 188.00 (143.02) | 76% | 13.37 (10.60) | 79% |
| 34 | 1.81 (0.01) | 1% | 14.50 (02.50) | 17% | 0.67 (0.14) | 21% |
| 35 | 2.30 (0.39) | 17% | 285.33 (142.13) | 50% | / | / |
| 36 | 2.41 (0.30) | 13% | 121.43 (59.04) | 49% | 5.56 (4.49) | 81% |
| 37 | 2.77 (0.19) | 7% | 372.69 (196.70) | 53% | / | / |
| 38 | 3.31 (0.44) | 13% | 79.67 (41.63) | 52% | 5.99 (3.33) | 56% |
| 39 | 1.34 (0.00) | 0% | 217.00 (00.00) | 0% | 2.17 (0.00) | 0% |
| 40 | 2.16 (0.32) | 15% | 374.00 (267.95) | 72% | 5.22 (4.08) | 78% |
| 41 | 2.35 (0.32) | 14% | 336.67 (143.83) | 43% | 21.84 (10.86) | 50% |
| 42 | 2.03 (0.06) | 3% | 41.00 (08.00) | 20% | 1.22 (0.37) | 31% |
| 43 | 3.27 (0.44) | 13% | 455.17 (255.83) | 56% | 14.47 (8.90) | 62% |
| 44 | 3.93 (0.35) | 9% | 204.94 (84.56) | 41% | 29.91 (13.17) | 44% |
| 45 | 4.53 (0.87) | 19% | 281.52 (206.77) | 73% | 7.35 (4.75) | 65% |
| 46 | 2.41 (0.38) | 16% | 23.25 (14.24) | 61% | 1.08 (0.89) | 82% |
| 47 | 3.80 (0.47) | 12% | 48.25 (28.36) | 59% | 1.42 (0.75) | 53% |
| 48 | 2.66 (0.17) | 6% | 99.22 (76.53) | 77% | 6.79 (5.13) | 76% |
| 49 | 0.90 (0.00) | 0% | 1.00 (00.00) | 0% | / | / |
| 50 | 2.74 (0.17) | 6% | 304.50 (144.29) | 47% | / | / |
| 51 | 2.75 (0.15) | 5% | 122.38 (67.02) | 55% | 5.23 (2.86) | 55% |
| 52 | 3.54 (0.81) | 23% | 274.12 (130.00) | 47% | 7.12 (3.76) | 53% |
| 53 | 4.45 (0.55) | 12% | 222.20 (149.39) | 67% | 10.68 (5.16) | 48% |
| 54 | 3.30 (0.18) | 6% | 937.11 (384.23) | 41% | 14.78 (5.29) | 36% |
| 55 | 3.90 (0.60) | 15% | 46.95 (24.02) | 51% | 2.84 (1.65) | 58% |
| 56 | 3.24 (1.12) | 35% | 10.25 (03.77) | 37% | 0.45 (0.27) | 59% |
| 57 | 6.87 (1.83) | 27% | 123.32 (82.65) | 67% | 3.72 (2.64) | 71% |
| 58 | 5.82 (1.19) | 20% | 216.60 (152.46) | 70% | 5.65 (4.52) | 80% |

a. Standard deviation.

b. Coefficient of variance.

c. Not applicable.

Appendix S2

- Puffs Per Day Calculation Rationale

Puffs per day at the population level can be calculated by averaging the number of puffs from the active users in each day. Specifically, the number of puffs for user *j* at day *k* was recorded by PR-ENDS and uploaded to the cloud. Due to the fact that different participant was enrolled into the observation session at different date, day 1 for user *j* was recognized as the first date in which user *j*’s puff data was observed. The puffs per day at the population level (for the active users) at day *k* was then calculated based on equation (2). It is noted that the number of active users at day *k* refers to the number of users whose puff data was observed at day *k*.

${Puffs Per day}_{@ Day k}= \frac{\sum_{j} {Number of Puffs}_{\mathrm{User}j @ Day k}}{{Number of Active Users}_{@ Day k}}$ (2)

With the puffs per day at day *k* calculated, the puffs per day over time was plotted in Figure 4(a). The standard error of puffs per day was calculated for the active users at day *k*.

- Puff Duration Per Day Calculation Rationale

Puff duration per day at the population level can be calculated by averaging the puff duration from the active users in each day. Specifically, the puff duration for user *j* at day *k* was calculated based on equation (3) with the PR-ENDS recorded data. Due to the fact that different participant was enrolled into the observation session at different date, day 1 for user *j* was recognized as the first date in which user *j*’s puff data was observed.

${Puff Duration}_{\mathrm{User}j @ Day k}= \frac{\sum_{i} {Puff Duration}_{\mathrm{Puff}i, User j @ Day k}}{{Number of Puffs}_{\mathrm{User}j @ Day k}}$ (3)

Puff duration per day at the population level (for the active users) at day *k* was then calculated based on equation (4). It is noted that the number of active users at day *k* refers to the number of users whose puff data was observed at day *k*.

${Puff Duration}_{@ Day k}= \frac{\sum_{j} {Puff Duration}_{\mathrm{User}j @ Day k}}{{Number of Active Users}_{@ Day k}}$ (4)

With the puff duration per day at day *k* calculated, the puff duration per day over time was plotted in Figure 4(b). The standard error of puff duration per day was calculated for the active users at day *k*.

- Nicotine Consumption Per Day Calculation Rationale

Nicotine consumption per day at the population level can be calculated by taking the nicotine emission per puff and the associated number of puffs from the active users in each day. The calculation of nicotine emission per puff was rationalized in Appendix S1 and the number of puffs from active users in each day was rationale in equation (2).


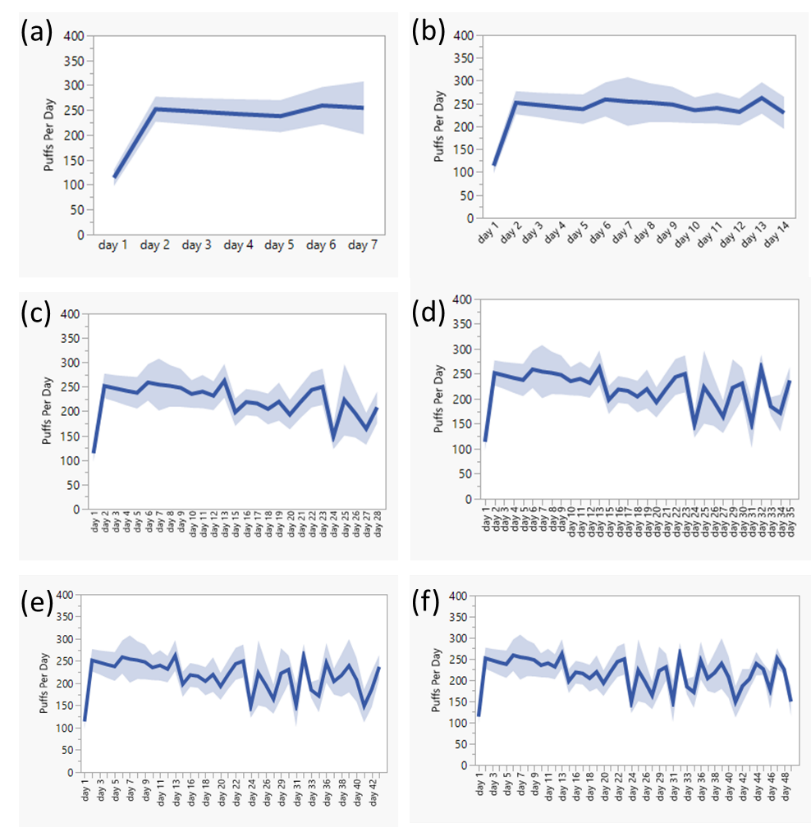


Figure S2 Observation of puffs per day during the actual use of PR-ENDS over (a) one, (b) two, (c) four, (d) five, (e) six, and (f) seven weeks. The line represents the average, and the band represents the standard error.

# Reference

Hensel, E. C., Eddingsaas, N. C., Saleh, Q. M., Jayasekera, S., Sarles, S. E., Thomas, M., Myers, B. T., DiFrancesco, G., & Robinson, R. J. (2021). Nominal Operating Envelope of Pod and Pen Style Electronic Cigarettes. *Front Public Health*, *9*, 705099. <https://doi.org/10.3389/fpubh.2021.705099>

Talih, S., Balhas, Z., Eissenberg, T., Salman, R., Karaoghlanian, N., El Hellani, A., Baalbaki, R., Saliba, N., & Shihadeh, A. (2015). Effects of user puff topography, device voltage, and liquid nicotine concentration on electronic cigarette nicotine yield: measurements and model predictions. *Nicotine Tob Res*, *17*(2), 150-157. <https://doi.org/10.1093/ntr/ntu174>
