# Supplementary figures and images for: Actual Use Behavior Assessment of a Novel Puff Recording Electronic Nicotine Delivery System: Observation Study
Source: JMIR Form Res. 2023 Feb 8;7:e43175. doi: 10.2196/43175 (PMC9947749; doi:10.2196/43175)

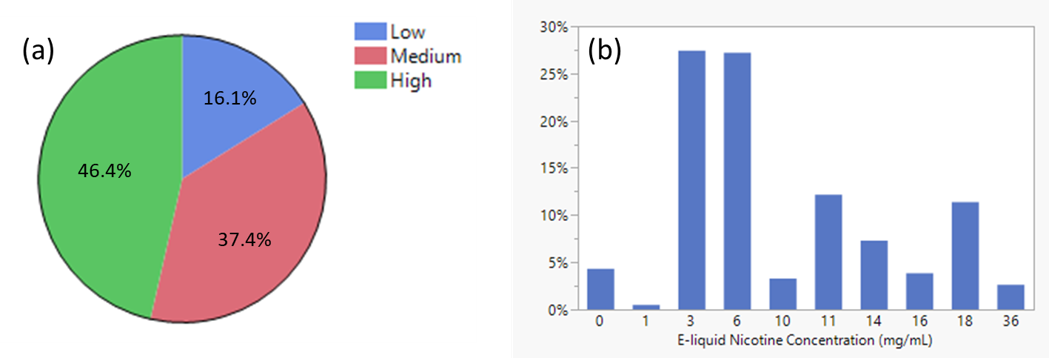

Supplement: Multimedia Appendix 2 [file formative_v7i1e43175_app2.png]
